# 维奈克拉联合去甲基化药物在不能耐受强化诱导治疗的初治核心结合因子相关急性髓系白血病患者中的疗效分析

**DOI:** 10.3760/cma.j.cn121090-20241209-00546

**Published:** 2025-08

**Authors:** 思懿 韩, 晓燕 徐, 萌 周, 海侠 周, 德沛 吴, 悦 韩

**Affiliations:** 苏州大学第一附属医院血液科，苏州 215006 National Clinical Research Center for Hematologic Diseases, Jiangsu Institute of Hematology, the First Affiliated Hospital of Soochow University, Suzhou 215006, China

**Keywords:** 核心结合因子白血病, 维奈克拉, 去甲基化药物, Core-binding factor leukemia, Venetoclax, Hypomethylating agents

## Abstract

**目的:**

探究维奈克拉（Ven）联合去甲基化药物（HMA）在不能耐受强化诱导治疗的核心结合因子相关急性髓系白血病（CBF-AML）中的疗效。

**方法:**

回顾性纳入2020年1月至2023年6月期间在苏州大学第一附属医院血液科接受Ven联合HMA作为诱导治疗的所有初治且年龄小于60岁的CBF-AML患者。收集患者的基本特征，比较分析患者的治疗反应。

**结果:**

共纳入70例初诊时采用Ven联合HMA诱导治疗方案的患者，男38例，女32例，中位年龄43（34～55）岁。首个化疗周期结束时，70例CBF-AML患者中共有44例（62.9％）达完全缓解或血细胞未完全恢复的完全缓解（CR/CRi），16例（22.9％）达部分缓解，10例（14.2％）未缓解。32例t（8;21）阳性的AML患者中仅有8例（25.0％）达到CR/CRi，其中3例患者（3/8，37.5％）可测量的残留病（MRD）仍为阳性；而38例inv（16）患者中，36例（94.7％）达CR/CRi，其中12例（12/36，33.3％）为MRD阳性。携带CBFß::MYH11融合基因的患者对Ven联合HMA诱导方案的应答率明显高于携带RUNX1::RUNX1T1融合基因的患者（*P*<0.01）。

**结论:**

Ven联合HMA作为一种新的治疗策略，在inv（16）患者中有显著的疗效，但在t（8;21）患者中缓解率相对较低。

## Abstract (en)

**Objective:**

To investigate the efficacy of venetoclax combined with hypomethylating agents（Ven-HMA）, in patients with core binding factor acute myeloid leukemia（CBF-AML）intolerant to intensive induction therapy.

**Methods:**

This study retrospectively analyzed patients newly diagnosed with CBF-AML who were aged <60 years and who received Ven-HMA as induction therapy at the Department of Hematology, the First Affiliated Hospital of Soochow University, between January 2020 and June 2023. Baseline characteristics and treatment responses of the patients were collected.

**Results:**

A total of 70 treatment-naïve patients receiving Ven-HMA induction therapy were enrolled, of which 38 were men and 32 women［median age: 43（34–55）years］. Of the 70 patients, 44（62.9％）achieved complete remission（CR）/CR with incomplete hematologic recovery（CRi）, 16（22.9％）achieved partial remission, and 10（14.2％）exhibited no response after one induction cycle. Among the 32 t（8;21）-positive patients with AML, only 8（25.0％）achieved CR/CRi, of whom 3（37.5％）remained measurable residual disease（MRD）-positive; among the 38 inv（16）-positive patients, 36（94.7％）achieved CR/Cri, of whom 12（33.3％）remained MRD-positive. Patients harboring the CBFβ::MYH11 fusion gene showed significantly higher response rates to Ven-HMA induction than those with the RUNX1::RUNX1T1 fusion gene（*P*<0.01）.

**Conclusion:**

Ven-HMA represents a novel therapeutic strategy that exhibits significant efficacy in inv（16）-positive patients; however, it demonstrates relatively lower remission rates in t（8;21）-positive patients.

t（8;21）（q22;q22）和inv（16）（p13;q22）/t（16;16）（p13;q22）是急性髓系白血病（AML）患者中常见的染色体异常，这类患者被统称为核心结合因子相关急性髓系白血病（CBF-AML）。对于身体状况良好的CBF-AML患者，采用阿糖胞苷联合蒽环类药物诱导化疗，随后用高剂量阿糖胞苷进行1～4个疗程的巩固治疗，预计5年生存率可达80％[1]–[3]。然而，在现实中，许多患者无法耐受传统强化化疗的不良反应。对于老年或身体状况不佳的患者，低强度治疗方案，如维奈克拉（Ven）联合去甲基化药物（HMA），已成为新的标准治疗方案之一。Ven是一种选择性BCL-2抑制剂，不仅可以增加AML细胞对去甲基化药物的敏感性[4]–[5]，还可以通过影响氨基酸的氧化磷酸化来靶向白血病干细胞（LSC）[6]–[8]。既往研究已证实，Ven联合HMA在老年和不适合强化化疗的AML患者中具有较高的缓解率[9]–[11]，但这些研究均未包括CBF-AML患者。本研究旨在探讨Ven联合HMA在CBF-AML治疗中的可行性。

## 病例与方法

1. 病例资料：本研究纳入了2020年1月至2023年6月期间在我中心接受Ven联合HMA诱导治疗的所有新诊断的、年龄小于60岁的CBF-AML患者。收集患者的年龄、性别、住院时间、初诊白细胞计数、骨髓细胞形态、免疫表型、细胞遗传学和分子遗传学、基因突变、第1周期诱导治疗结束后患者疾病缓解情况等临床资料。诊断标准：AML的诊断符合文献[12]标准，通过标准核型检查发现t（8;21）（q22;q22）或inv（16）（p13;q22）染色体异常，或通过实时定量聚合酶链反应（RT-qPCR）或荧光原位杂交（FISH）检测到RUNX1::RUNX1T1或CBFß::MYH11融合转录本，即可确诊为CBF-AML。排除标准：继发性AML患者，如骨髓增生异常综合征或潜在骨髓增殖性肿瘤转化AML患者，急性早幼粒细胞白血病患者，以及未接受治疗或接受其他治疗的AML患者。

2. 治疗方案：患者接受阿扎胞苷75 mg/m²×7 d，或地西他滨20 mg/m²×5 d，Ven 100 mg第1天，200 mg第2天，400 mg第3～28天，Ven的目标剂量为400 mg/d，但如与CYP3A抑制剂合用，则根据建议减少剂量。

3. 疗效及不良反应评价：根据2022年欧洲白血病网（ELN）指南[13]，疗效定义如下。完全缓解（CR）：骨髓原始细胞<5％，外周血未见原始细胞，无髓外病变，中性粒细胞绝对计数≥1.0×10^9^/L且PLT≥100×10^9^/L；CR伴血细胞不完全恢复（CRi）：骨髓原始细胞<5％，外周血未见原始细胞，无髓外病变，中性粒细胞绝对计数<1.0×10 ^9^/L或者PLT<100×10^9^/L；部分缓解（PR）：血细胞恢复达CR，骨髓原始细胞比例5％～25％，且较治疗前下降至少50％。未缓解（NR）：评估不符合CR、CRi、PR。通过RT-qPCR监测可测量的残留病（Measurable residual disease, MRD），根据ELN指南，CD45目标免疫表型表达细胞小于0.1％定义为MRD阴性。不良反应评价依据美国国家癌症研究所常见不良反应事件评价标准[14]进行评价。

4. 统计学处理：采用统计学软件R 4.2.0进行统计分析，分类变量用频数（百分比）描述，采用秩和检验和Fisher确切概率检验进行两组比较。符合正态分布的连续变量用“均数±标准差”表示，采用*t*检验进行比较。不符合正态分布的连续变量用中位数（范围）描述，两组间采用Mann-Whitney *U*检验比较。计数资料采用频率表示，组间的差异比较采用卡方检验。*P*<0.05为差异有统计学意义。

## 结果

1. 一般资料：本研究共纳入了70例患者。患者不适合接受强化治疗的原因包括活动性感染、败血症、肝肾或心脏功能障碍等。我们收集了患者初诊时的血红蛋白、白细胞计数、血小板计数、骨髓原始细胞以及二代测序结果等数据。与t（8;21）阳性患者相比，inv（16）阳性患者在初诊时WBC更高（*P*＝0.039）、伴有NRAS突变的比例较高（50.0％对3.1％，*P*<0.001），而t（8;21）阳性患者则伴有更高频率的ASXL2突变（21.9％对2.1％，*P*＝0.002）。70例患者的基线特征详见表1。

**表1:** 70例接受维奈克拉联合去甲基化药物治疗的初治核心结合因子相关急性髓系白血病患者基本信息

特征	t（8;21）组 （32例）	inv（16）组 （38例）	*P*值
性别（例）			0.200
男	14	24	
女	18	14	
年龄［岁，*M*（范围）］	38（34～44）	42（37～55）	0.260
WBC［×10^9^/L，*M*（范围）］	8（4～19）	21（6～46）	0.039
HGB［g/L，*M*（范围）］	78（60～97）	78（65～108）	0.600
PLT［×10^9^/L，*M*（范围）］	36（17～75）	36（19～86）	0.500
骨髓原始幼稚细胞［％，*M*（范围）］	49（25～69）	60（29～69）	0.400
染色体核型（例）			0.718
简单核型	25	31	
复杂核型	7	7	
KIT突变（例）			0.268
阴性	16	24	
阳性	16	14	
FLT3-ITD突变（例）			0.900
阴性	24	28	
阳性	8	10	
KRAS突变（例）			0.130
阴性	30	31	
阳性	2	7	
NRAS突变（例）			<0.001
阴性	31	19	
阳性	1	19	
PTPN11突变（例）			0.859
阴性	30	32	
阳性	2	4	
ASXL2突变（例）			0.002
阴性	25	37	
阳性	7	1	
RAD21突变（例）			0.456
阴性	30	37	
阳性	2	1	
CSF3R突变（例）			0.053
阴性	27	37	
阳性	5	1	
TET2突变（例）			0.281
阴性	28	36	
阳性	4	2	

2. 疗效分析：在第1个诱导化疗周期结束时，70例CBF-AML患者中共有44例（62.9％）患者达CR/CRi，16例（22.9％）患者达PR，10例（14.2％）患者NR。t（8;21）阳性AML患者组中仅8例（8/32，25.0％）患者达到CR/CRi，15例（15/32，46.9％）患者达PR，9例（9/32，28.1％）患者NR，3例（3/8，37.5％）患者MRD阳性；inv（16）阳性AML患者组中，36例（36/38，94.7％）达到CR/CRi，1例（1/38，2.6％）患者达PR，1例（1/38，2.6％）患者NR，12例（12/36，33.3％）患者MRD阳性。inv（16）阳性AML患者对Ven联合HMA方案应答率更高（*P*<0.01）。

我们的队列中有30例（42.9％）患者伴有KIT突变，其中t（8;21）阳性患者16例，inv（16）阳性患者14例。在伴有KIT突变的t（8;21）患者中，仅有3例（18.8％）在1个化疗周期后达到CR/CRi，而14例伴有KIT突变的inv（16）患者均达到CR/CRi。对于伴有FLT3-ITD突变的患者，8例t（8;21）AML患者中仅2例（25.0％）在1个化疗周期后达到CR/CRi，而10例inv（16）患者均达CR/CRi，差异均具有统计学意义（均*P*<0.05）。

3. 不良反应：所有接受Ven联合HMA方案治疗的患者中，最常见的非血液学不良反应是感染，包括肺部感染（21/70，31.4％）和肛周感染（3/70，4.3％），其次为胃肠道反应，包括恶心和呕吐（13/70，18.6％）、便秘（12/70，17.1％）、腹泻（5/70，7.1％）。血液学不良反应方面，3～4级白细胞减少、血小板减少和贫血的发生率分别为44.3％（31/70）、14.3％（10/70）和18.6％（13/70）。

## 讨论

CBF-AML患者对传统强化诱导方案往往能有较好的反应，然而，仍有许多患者无法耐受传统强化诱导方案的不良反应。先前一项Ⅰb期研究结果显示，对于不适合强化化疗的AML患者，与仅接受HMA治疗的患者相比，接受Ven联合HMA的患者总生存期更长，缓解率更高[15]–[17]。但这些临床研究大部分未包括CBF-AML患者或样本数量较小，因此，该联合治疗方案在CBF-AML患者中的潜在效果和应用前景仍需进一步探讨。

根据既往研究结果，伴有NPM1或IDH2突变的AML患者缓解率更高且缓解期更长[18]–[19]，而伴有信号转导通路相关突变的患者，如FLT3、RAS、TP53等，对以Ven为基础的治疗方案更易产生适应性耐药[20]–[21]。于文静等[22]报道了6例初治t（8;21）AML患者，接受Ven联合HMA方案治疗后疗效评估达CR/CRi者仅2例；6例难治/复发AML患者，接受Ven联合HMA方案治疗后疗效评估无一例达CR/CRi，其中伴有KIT突变患者5例。KIT突变作为CBF-AML患者中影响预后的独立不良因素，其持续表达也与化疗耐药和复发有关[23]–[26]，这与我们的研究结果一致。在我们的研究中，16例伴有KIT突变的t（8;21）患者中，仅有3例（18.8％）在1个化疗周期后达到CR/CRi，表明在此类患者亚组中应谨慎使用Ven联合HMA诱导治疗方案，但在inv（16）患者中，14例伴有KIT突变患者均达到CR/CRi，说明对于此类患者，即使在伴发KIT突变的情况下，Ven联合HMA仍可作为inv（16）患者有效的诱导治疗方案。米瑞华等[27]报道了12例以Ven为基础的方案治疗伴t（8;21）的AML患者，结果显示，在5例接受Ven联合HMA方案治疗的初治患者中，1个疗程结束后评估4例患者NR，仅1例患者达CRi，而在此基础上联合高三尖杉酯碱（HHT）1 mg/m^2^第1～7天，可提高疗效。这提示我们在使用Ven联合HMA方案治疗t（8;21）AML患者时，联合其他药物使用或许可提高此类患者的应答率，在临床工作中值得我们进一步探索。

尽管t（8;21）和inv（16）AML被统称为CBF-AML，在临床治疗中也采用类似的治疗方案，实际上，t（8;21）和inv（16）AML应被视为不同的疾病。t（8;21）AML虽然预后良好，但仍有30％～40％的患者在后续治疗中复发，长期预后较inv（16）AML患者差，该类型更常与表观遗传调控因子、染色质修饰因子和凝聚蛋白的突变相关。相反，inv（16）AML则更常与激酶突变相关[28]–[29]。研究发现，转录因子RUNX1::RUNX1T1融合基因可通过诱导BCL-2启动子处的抑制性染色质构象而产生表观遗传沉默，t（8;21）AML患者的BCL-2表达较低，且线粒体形态发生途径下调[30]–[32]，这可能是Ven联合HMA的治疗方案在t（8;21）AML患者中的疗效不如预期的原因之一。后续研究中我们应该更多地关注此类患者，以探索更有效的治疗方案。

总之，Ven联合HMA的诱导治疗方案在t（8;21）AML患者中的疗效较inv（16）患者差。本研究为不适合接受强化诱导治疗的CBF-AML患者使用Ven联合HMA以及后续治疗策略提供了新的见解，由于样本量较小且为回顾性研究，结果可能存在一定偏倚，需进一步开展更大样本量的研究来验证。
